# Usefulness of screening tools in the evaluation of long-term effectiveness of DREZ lesioning in the treatment of neuropathic pain after brachial plexus injury

**DOI:** 10.1186/s12883-014-0225-9

**Published:** 2014-12-09

**Authors:** Pavel Haninec, Radek Kaiser, Libor Mencl, Petr Waldauf

**Affiliations:** Department of Neurosurgery, Third Faculty of Medicine, Charles University, Hospital Kralovske Vinohrady, Srobarova 50 100 34, Prague, Czech Republic; Department of Anesthesiology and Critical Care Medicine, Third Faculty of Medicine, Charles University, Hospital Kralovske Vinohrady, Prague, Czech Republic

**Keywords:** DREZ lesioning, Deafferentation pain, Screening tool, Neuropathic pain, Brachial plexus injury

## Abstract

**Background:**

Despite high success rate of DREZ lesioning in the treatment of intractable central pain, there is still a significant incidence of patients without satisfactory post-operative effect. The aim of the study was to evaluate the long-term effect of DREZ lesioning using both a subjective assessment using a visual analog scale (VAS) to quantify residual pain and an assessment using the screening tool (painDETECT Questionnaire, PD-Q).

**Methods:**

DREZ lesioning was performed in 52 patients from a total 441 cases with brachial plexus injury (11.8%) during a 17-year period (1995–2011). The effect of surgery was retrospectively assessed in 48 patients.

**Results:**

A decrease in pre-operative pain by more than 75% (Group I) was achieved in 70.8% of patients and another 20.8% reported significant improvement (Group II). The surgery was unsucessful in 8.4% (Group III). We found a significant correlation between ‘improvement’ groups from both methods of assessments. Patients from Group I usually complained of residual nociceptive pain according to PD-Q, patients from Group II typically had pain of unclear origin, and all cases those in Group III suffered from neuropathic pain, Cramer’s V = .66, P < .001. Overall, 66.7% of patients had resolved neuropathic pain, 20.8% patients had more serious complaints and may also suffer from residual neuropathic pain, while 12.5% had unresolved neuropathic pain.

**Conclusion:**

DREZ lesioning is a safe and effective method with success rates of about 90%. PD-Q scores correspond to subjective satisfaction with the surgery and it seems to be a suitable screening tool for finding patients with residual neuropathic pain after surgery.

## Background

Pain is an early symptom in up to 70% of patients with brachial plexus injury. In up to 20% of cases severe intractable pain develops. Persistent pain with sporadic acral irritations described by patients as cutting or burning, is typical [1,2]. In 90%, the pain corresponds to the avulsion of one of the lower roots. Pain does not appear at the time of injury, but typically several days after. Pathophysiology of the pain is not fully clarified, but it originates after the loss of sensory impulses from the periphery which leads to the creation of pathologic pain generator in the dorsal horn of the spinal cord, in Rexed’s lamina I [3]. Irritations are treatable with standard analgesics in the early stages and often surcease with the restoration of sensory innervation of the median nerve. Although central neuropathic pain is often refractory, pharmacotherapy may give satisfactory or even good relief. Worth trying are the gabapentinoids, tricyclic antidepressants, tramadol, and for partial injuries, lamotrigine [4,5]. However, if pain treatment is inadequate and pain progresses it indicates that a central component is present. The only causal therapy is in these situations is DREZ (dorsal root entry zone) thermocoagulation (DREZ-T) [2,3,6-10], which was first described by Nashold [8]. This method can also be used for trigeminal or post-herpetic neuralgia or in pain after spinal cord trauma, but results are generally not as favorable [11].

To our knowledge, no study has evaluated residual neuropathic pain or success of DREZ-T using a screening tool. The painDETECT Questionnaire (PD-Q) was developed in Germany for use in individuals with back pain and incorporates an easy to use patient-based (self-report) questionnaire that does not require a clinical examination [12].

The aim of this study was to evaluate the long-term effect of DREZ-T in patients treated for severe neuropathic pain that developed after brachial plexus injury. The evaluation used both a subjective assessment using a visual analog scale (VAS) to quantify residual pain and an assessment using the PD-Q score [12]. Additionally, we wanted to test the usefulness of the screening tools in evaluating residual neuropathic pain in such patients.

## Methods

A total of 52 DREZ thermocoagulations have been performed, by the senior author, during the period 1995 – 2011 (P. H., Dept. of Neurosurgery, General Military Hospital, Prague, since 1998 present department). Surgical procedures were completed on 48 men and four women with an average age of 40 years (SD = 9.6, range 21 – 70). Twenty-nine cases were localized on the right and 23 on the left side. Patient data are summarized in Table 1.

**Table 1:** **Group of patients undergoing DREZ thermocoagulation**

**Patient**	**Age (yrs), Sex**	**Number of lesions**	**Complications**	**Effect**	**PD-Q score**	**Follow-up (mos)**
1	27, m	24	-	2	6	170
2	45, f	25	-	1	14	156
3	28, m	32	-	1	6	140
4	45, m	19	-	-	-	D
5	51, m	25	-	1	12	110
6	25, m	28	-	2	15	169
7	23, m	39	Motor	2	6	167
8	47, m	22	-	-	-	N/A
9	50, m	15	-	3	33	155
10	47, m	29	-	1	6	178
11	31, m	45	-	2	15	150
12	35, m	25	-	1	4	139
13	22, m	25	-	1	10	180
14	70, m	31	-	1	6	120
15	45, f	21	-	2	18	113
16	27, m	33	Motor	2	7	115
17	25, m	29	-	1	8	159
18	38, m	42	Sensory	1	12	126
19	40, m	25	-	1	9	108
20	48, m	40	-	1	5	105
21	40, m	32	-	1	5	100
22	39, m	29	-	-	-	D
23	50, m	20	-	-	-	N/A
24	29, m	47	Sensory, Motor	1	5	82
25	40, m	38	-	1	9	76
26	58, m	41	-	1	8	70
27	35, m	16	-	2	15	68
28	55, m	34	-	1	7	67
29	38, m	48	-	1	0	67
30	31, m	50	-	3	30	63
31	35, m	25	-	1	5	62
32	51, m	26	-	1	12	61
33	24, m	45	-	2	21	60
34	47, m	22	Sensory	1	9	59
35	40, m	18	-	1	10	58
36	57, m	29	-	1	5	57
37	49, m	20	-	3	24	51
38	29, m	37	-	1	16	50
39	28, m	26	-	1	13	49
40	51, m	44	-	1	17	48
41	32, m	25	Sensory	1	8	47
42	45, m	16	-	2	16	46
43	28, m	40	-	1	2	45
44	42, f	37	-	1	5	44
45	33, m	29	Sensory	1	6	37
46	33, m	39	-	3	29	36
47	41, m	49	Sensory	2	25	33
48	21, m	39	-	1	4	32
49	29, m	28	-	1	4	31
50	55, m	25	-	1	14	28
51	34, m	33	-	1	5	26
52	20, f	34	-	1	6	26

M, f – male, female. Effect – intensity of preoperative pain, 1 = Group I, 2 = Group II, 3 = Group III – see Table 2. PD-Q score – assessed by painDETECT Questionnaire. D – patient deceased, N/A – patient not available for follow-up examination.

The study was approved by the hospital institutional review board and informed consent to participate in the study and for the publication of individual clinical details was obtained from each patient.

### Indication criteria

Patients were indicated for DREZ-T after development of severe, unbearable pain in the affected extremity after all types of analgesia (including high dose of opioids, anticonvulsants and tricyclic antidepressants) had been tried and found to be inadequate. The vast majority of procedures were performed before a planned reconstruction of the brachial plexus. All patients underwent preoperative EMG examination using needle concentric electrodes and nerve conduction studies. CT myelography showed avulsion of minimally two cervical roots in all patients.

### Operative technique

The procedures were performed in the semi-sitting position with the head fixed in three-point fixation after shaving the occipital area. An incision was made vertically from external occipital protuberance to the vertebra prominens. Multilevel hemilaminectomies (2–4) were performed to expose the spinal cord at the level of the nerve root avulsion. Laminectomy and durotomy were extended until finding the nearest outgoing posterior root. The lesions were made after DREZ localization (see below), using a radiofrequency electrode with a tip depth 2 mm under the spinal cord surface. Lesioning time was 15 s, lesioning temperature was 75°C; lesioning device was a Radionics RFG-3C Plus (Valleylab, Colorado, USA).

The strip electrode with two active members (each 1 mm in diameter, 5 mm distance between them) was slipped under the dura at the rostral end of exposed spinal cord. Responses were amplified at a gain setting of 100 with the high frequency filter set at 5 kHz and the low frequency filter set at 20 Hz. Responses were not averaged (Dantec Counterpoint 2, Minneapolis, USA). The stimulating electrode was a bipolar stimulating electrode with constant distance between tips of 1 mm. A 200-μsec square-wave impulse and a stimulation rate of 3 stimuli per second were used.

Initially the stimulating electrode was placed over the dorsal column. The distance between stimulating and recording electrodes varied from 3 to 8 cm. The intensity of the stimulus was gradually increased until an evoked potential with amplitude of approximately 30 – 50 uV was elicited. The stimulus intensity was not changed during the rest of procedure and ranged from 0.1 to 0.9 mA. Using an operating microscope, the neurosurgeon gradually stimulated dorsolateral surface of spinal cord at a constant distance from the registration electrode, in approximately 2 mm steps. Sites where the stimulation electrode failed to evoke a response were considered DREZ and a thermocoagulation lesion was made at that site. The stimulating technique was repeated along the long axis of the spinal cord at 1 cm intervals. Our operative technique was previously published [10].

### Postoperative examination

All patients were hospitalized for one week. Patients were examined clinically every half year. Minimum time of follow-up was two years. The last evaluation was a clinical examination at our outpatient clinic in the more recent cases or via mail in patients who underwent surgery more than five years ago. At first, the residual pain was assessed using VAS with the present-day score counted as a percentage decrease from the original level. Based on these scores, patients were divided into three groups according to intensity of residual pain (Table 2). Next the patients were assessed using the official Czech version of the painDETECT Questionnaire (PD-Q) [12]. This screening tool was chosen for its simplicity and because it does not require a clinical examination.

**Table 2:** **Details of post-surgery residual pain**

	**Decrease of preoperative pain**	**Number of patients (%)**	**Average PD-Q score**	**Screening results No : Un : Ne (%)**
Group I	> 75%	34 (70.8%)	7.85 (SD = 3.25, range 0–17)0*	29 (85.3% #) : 5 (14.7%) : 0
Group II	75 - 50%	10 (20.8%)	14.4 (SD = 4.8, range 6–25)*	3 (30%) : 5 (50% #): 2 (20%)
Group III	< 50%	4 (8.4%)	29 (SD = 2.5, range 24–33)*	0 : 0 : 4 (100% #)

Decrease of preoperative pain – percentage decrease of preoperative VAS, PD-Q score - painDETECT Questionnaire score, Screening results: No – Nociceptive - a total score of ≤12, a neuropathic pain component is unlikely, Un – result is unclear, a neuropathic pain component could be present, Ne – Neuropathic, a score of ≥19, a neuropathic component is likely, Number of cases and (%) – percentage of cases within main group. * - statistically significant difference between all three groups (P < .001, ANOVA with post-hoc analysis using the Fisher LSD test), # - statistically significant correlation between adequate groups (Group I – No, Group II – Un, Group III – Ne) (Cramer’s V = .66, Yates’Chi-square P < .001).

### Statistical analysis

The results were analyzed using ANOVA with post-hoc analysis using the Fisher LSD test (for continuous data) and Cramer’s V and Yates Chi-square test (for categorical data). Analyses were done using Statistica 10.0 software (StatSoft Inc., Tulsa, Oklahoma, USA) and R (http://www.r-project.org). Significance was assumed at p <0.05.

## Results

Thermal lesions were performed over the entire range of avulsions in all patients, the average number of lesions was 31 (SD = 7.9, range 15 – 50). Fifty-one patients had procedures before or just after reconstruction of the brachial plexus; one man was successfully treated 28 years after the original accident. Of the 441 patients undergoing a brachial plexus reconstruction, the thermal lesion group represented 11.8%. Of 52 patients treated with thermal lesions, we were able to obtain data regarding the subjective assessment of their condition in 48 cases. Two men died of unrelated causes and another two patients could not be contacted for the final follow-up examination.

A decrease in pre-operative pain intensity of more than 75% was considered a definite success. Based on our follow-up, this goal was achieved in 70.8% of patients. Another 20.8% reported significant improvement, but with some pain persistence, usually in the form of dull pain or paresthesias of the affected upper limb. Overall satisfaction with the surgery was achieved in 91.6% of patients. The surgery was considered to be unsuccessful in the remaining four cases (8.4%). In two cases, after improvement in the early post-operative period, progressive worsening to the original level began at one and three months post-surgery. The two other cases reported persistence of very severe dull pain. In one case the patient decided to amputate the affected limb; however, this also failed to resolve the issue (No. 30).

**Figure 1:**
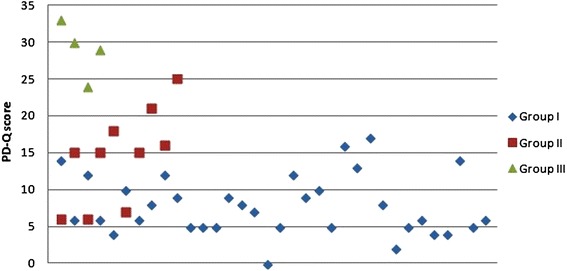
**PainDETECT Questionnaire score (PD-Q score) in all three groups.** For details see Table 2.

The PD-Q score was in agreement with subjective assessments of residual pain: the average pain score in Group I (7.85) was statistically significantly lower than in Group II (14.4, p <0.001). Both are significantly lower than in Group III (29, p <0.001). We found a statistically significant correlation between adequate-treatment groups (Group I – Nociceptive pain, Group II – Unclear, Group III – Neuropathic pain) (Cramer’s V 0.66, p <0.001). It was observed that 32/48 (66.7%) patients lost neuropathic pain, 10/48 (20.8%) patients had more serious complaints and may ultimately suffer from residual neuropathic pain while 6/48 (12.5%) had neuropathic pain. Details are summarized in Table 2 and the values are shown in Figure 1.

Post-operative complications occurred in 8 (15.4%) patients. In five cases, sensory deficits (4× hypoesthesia of ipsilateral lower extremity, 1× hemihypoesthesia of the trunk and lower limb) occurred. Motor leg weakness occurred in two patients and one case had a combined disability. Excluding the patient with hemihypoesthesia, in which there was a partial restoration of body sensitivity, all other patient complaints resolved and returned to pre-operative conditions.

## Discussion

The success and safety of DREZ-T in the treatment of intractable central pain caused by cervical root(s) avulsion have been confirmed repeatedly [2,6-10,13-15]. Generally, an immediate loss of pain can be achieved in 60 to 80% of cases, but the percentage decreases as time-after-accident increases. Some studies have found that the pain returns to some degree after several years in up to one-fifth of patients [2,13,14]; however, this was not confirmed by our study. Severe pain returned after several months only in two cases from Group III, none of the other patients reported any significant worsening or change of the quantity of residual pain over time. Some patients reported a change of quality of pain or irritations, but the change did not have a significant impact on their opinion regarding the effectiveness of the surgery.

An operative painkilling surgery is generally required in 10 to 15% of patients with spinal cord root avulsion [16,17]. Chen et al., for example, found that in a group of more than 500 patients, 60 DREZ thermocoagulations were performed (~12%) [13]. Our set of 11.8% corresponds to foreign experiences [13,18].

We divided patients into three groups (Table 2) according to Aichaoui et al.: Group I corresponds to VAS reduction up to 75%, these patients do not require supplemental analgesics, Group II – VAS reduction was between 50 and 75%, patients sometimes take analgesics and retrospectively would still agree to have the surgery, and Group III, which corresponds to VAS decrease less than 50%, such patients usually take painkillers (WHO Class II or opioids) and retrospectively would not undergo the surgery again.

It has been stated that there is no correlation between the number of roots avulsed or the extent of the DREZ-T procedure performed and the degree of pain reduction [17]. The best results have been achieved in sporadic irritations while persistent dull pain had a worse prognosis and higher tendency to recur [1,19]. This is in accordance with our study - all 10 patients from Group II had residual dull pain and only two from Group III had the same pain as before the surgery, while another two were relieved of only the strongest paroxysmal irritations. The results show that paroxysmal pain was successfully eliminated in 91.6% while severe dull pain was treatable in 70.8% cases. Patients from Group I usually reported only paroxysmal paresthesias or mild paroxysmal dull pain; some of them realized, for the first time during testing, that they still had some sensory disturbances. Our results are also parallel those presented in the broader literature [1,2,13,14].

The PD-Q was validated in a group of 392 individuals with nociceptive and neuropathic pain. Of its nine items, seven relate to sensory responses and two relate to temporal and spatial characteristics of the pain pattern. A total score ≤12 indicates that a neuropathic pain component is unlikely, whereas a score of ≥19 indicates that a neuropathic component is likely. Between these values the result are uncertain and a neuropathic pain component could be present. It has more than 80% sensitivity, specificity and positive predictive accuracy in the diagnosis of a neuropathic component in patients with low back pain [12]. This scale was used for evaluation of neuropathic pain in patients with spinal cord injuries with 68% sensitivity, 83% specificity and 78% diagnostic accuracy [20]. It can be also used for other types of neuropathic pain [21]. We used this screening tool because many of our patients live far from our department and were not inclined to travel to our facility for a clinical assessment so many years after surgery.

If we compare the success rate using a VAS decrease and the PD-Q scale, the results are similar (p <0.001): 70.8% (Group I): 66.7% (nociceptive pain), 20.8% (Group II): 20.8% (unclear pain) and 8.4% (Group III): 12.5% (neuropathic pain). The small differences probably result from (1) individual subjective assessment of pain intensity or (2) even if pre-operative pain intensity decreased dramatically, it is still possible that the patient has residual neuropathic pain. Our results suggest two clear conclusions about residual pain in our patients: (1) patients who are very satisfied with the effect of surgery (Group I) most often suffered from (obvious) nociceptive pain (85.3%) and (2) patients, in whom the surgery failed, had suffered from (obvious) neuropathic pain (100%, p <0.001). Patients in Group II, who were partially satisfied with the effectiveness of the surgery, might very well, suffer from a combination of nociceptive and neuropathic pain. The presence of nociceptive pain after brachial plexus injury seems to be unexpected. However, it may originate in the areas of preserved nerve supply in incomplete injuries or develop later in the areas reinnervated by neurotization. We can therefore conclude that it would be useful to evaluate patients who were not fully satisfied with the effectiveness of the DREZ-T surgery, using a screening tool like the PD-Q, which could reveal a potential neuropathic component of their residual pain. In such cases, a more effective conservative treatment would be indicated.

The risk of this procedure includes potentially serious neurological complications. The close proximity of corticospinal tract laterally and dorsal column medial lemniscal tract dorso-medially creates a risk motor failure of the ipsilateral lower extremity or sensitivity failure from the point of damage with lower limb ataxia, respectively. Anatomical and functional localization of DREZ is therefore essential for overall success. With careful monitoring, finding of vertical zero line is not usually difficult even though it is known that the distance from the midline to the DREZ can be quite variable [22]. Problems tend to occur in cases involving multiple avulsions with the presence of pseudomeningocele and dural scarring. They can cause changes in spinal anatomical arrangement of the surface. Needless to say, such delicate procedures require masterful execution; additionally, the radiofrequency electrode must be inserted to maximum depth of 2 mm and must be completely perpendicularly to the surface of the spinal cord and must be used in compliance with prescribed parameters (75°C, 15 seconds) [6,7,10]. The incidence of complications is different for different authors, ranging from 0 to 60% [18]. In our group, the frequency of complications was 15.4%.

Recent information has clouded the issue of deafferentation pain and made it a little less simple than previously thought. Deafferentation pain probably does not occur in children (both older with traumatic injuries, and infants with perinatal injuries) [23]. Aly et al. reported that electrical motor cortex stimulation is more effective for continuous than paroxysmal pain after brachial plexus injury [24]. The complete disappearance of pain after successful reinnervation [22] or only after endoneurolysis or neuroma removal [25] has been described. According to Bertelli et al., not all pain should be considered as deafferentation pain. They observed that in 400 patients, following rhizotomy performed for spasticity, deafferentation pain did not develop [3]. Other work from Bertelli suggests that the pain is not caused (or at least not always) by the avulsion, but is generated by the root(s), which remained intact. In 80% of patients with at least one well-preserved root, the pain subsided within three weeks after grafting. Another group, pain from an old injury was eliminated after selective anesthesia of preserved root using a CT-navigated technique [26]. The most recent study published by this author shows that pain occurs most frequently in complete brachial plexus palsy (84%) and, more interestingly, in cases where the avulsion of C8-Th1 was the only injury, deafferentation pain was never present [27]. Bonilla et al. described similar results with the subsidence of irritations after plexus element neurolysis or reconstruction, with pain subsiding in 78% of cases [6]. These studies are, however, not confirmed by published DREZ-T results [8,14] or by the case report of a man who was still pain free 26 years after the procedure [28] or by our current results. We think DREZ thermocoagulation is still a very effective method for treatment of severe neuropathic pain that can develops in some patients with supra-ganglion brachial plexus injury.

## Conclusion

We conclude that approximately 90% of our patients were satisfied with the effectiveness of DREZ thermocoagulation for intractable deafferentation pain after a brachial plexus injury.
